# Modulation of Gut Microbiome Community Mitigates Multiple Sclerosis in a Mouse Model: The Promising Role of *Palmaria palmata* Alga as a Prebiotic

**DOI:** 10.3390/ph16101355

**Published:** 2023-09-25

**Authors:** Shimaa Mohammad Yousof, Badrah S. Alghamdi, Thamer Alqurashi, Mohammad Zubair Alam, Reham Tash, Imrana Tanvir, Lamis AbdelGadir Kaddam

**Affiliations:** 1 Department of Physiology, Faculty of Medicine in Rabigh, King Abdulaziz University, Jeddah 21589, Saudi Arabia; lkaddam@kau.edu.sa; 2 Department of Physiology, Faculty of Medicine, Suez Canal University, Ismailia 41522, Egypt; 3 Neuroscience Unit, Department of Physiology, Faculty of Medicine, King Abdulaziz University, Jeddah 21589, Saudi Arabia; 4 Preclinical Research Unit, King Fahd Medical Research Center, King Abdulaziz University, Jeddah 21589, Saudi Arabia; 5 Faculty of Medicine in Rabigh, Pharmacology Department, King Abdulaziz University, Jeddah 21589, Saudi Arabia; talqurashi@kau.edu.sa; 6 Pre-Clinical Research Unit, King Fahad Medical Research Center, King Abdulaziz University, Jeddah 21589, Saudi Arabia; mzalam@kau.edu.sa; 7 Department of Medical Laboratory Sciences, Faculty of Applied Medical Sciences, King Abdulaziz University, Jeddah 21589, Saudi Arabia; 8 Department of Anatomy, Faculty of Medicine in Rabigh, King Abdulaziz University, Jeddah 21589, Saudi Arabia; rtash@kau.edu.sa; 9 Department of Anatomy, Faculty of Medicine, Ain Shams University, Cairo 3753450, Egypt; 10 Department of Pathology, Faculty of Medicine in Rabigh, King Abdulaziz University, Jeddah 21589, Saudi Arabia; ozafar@kau.edu.sa; 11 Physiology Department Faculty of Medicine, Alneelain University, Khartoum 11211, Sudan

**Keywords:** multiple sclerosis, gut–brain axis, red marine algae, *Palmaria palmata*, SCFA, gut microbiome, prebiotics

## Abstract

Background: Red marine algae have shown the potential to reduce inflammation, influence microbiota, and provide neuroprotection. Objective: To examine the prebiotic properties of *Palmaria palmata* aqueous extract (*Palmaria p.*) and its potential as a neuroprotective agent in multiple sclerosis (MS). Methods: eighty-eight adult Swiss mice were divided into four male and four female groups, including a control group (distilled water), *Palmaria p.*-treated group (600 mg/kg b.w.), cuprizone (CPZ)-treated group (mixed chow 0.2%), and a group treated with both CPZ and *Palmaria p.* The experiment continued for seven weeks. CPZ treatment terminated at the end of the 5th week, with half of the mice sacrificed to assess the demyelination stage. To examine the spontaneous recovery, the rest of the mice continued until the end of week seven. Behavioral (grip strength (GS) and open field tests (OFT)), microbiome, and histological assessments for general morphology of corpus callous (CC) were all conducted at the end of week five and week 7. Results: *Palmaria p.* can potentially protect against CPZ-induced MS with variable degrees in male and female Swiss mice. This protection was demonstrated through three key findings: (1) increased F/B ratio and expansion of the beneficial Lactobacillus, Proteobacteria, and Bactriodia communities. (2) Protection against the decline in GS induced by CPZ and prevented CPZ-induced anxiety in OFT. (3) Preservation of structural integrity. Conclusions: Because of its propensity to promote microbiota alterations, its antioxidant activity, and its content of −3 fatty acids, *Palmaria p.* could be a promising option for MS patients and could be beneficial as a potential probiotic for the at-risk groups as a preventive measure against MS.

## 1. Introduction

Neurodegenerative disorders are a class of pathologies distinguished by permanent demise, which results in a progressive and debilitating decline in nervous system functions [1]. There are variances in the frequency and clinical courses of MS patients, but the reasons behind these disparities are unexplained. MS is at least twice as common in women as in males. Women are more likely to obtain a benign relapsing–remitting variety of MS, but males are more likely to have a progressive course with more severe impairment and a shorter period till death [2].

The cuprizone (bis-cyclohexanone oxaldihydrazone) model is a demyelination and remyelination model. In the late 1960s, cuprizone-induced demyelination was first seen in Swiss mice [3]. Cuprizone is a copper chelator that produces demyelination in the mouse central nervous system (CNS), mainly in the corpus callosum (CC), by triggering oligodendrocyte death and activation of microglia and astrocytes inside demyelinating lesions [4]. Demyelination appears three weeks after the commencement of the cuprizone administration. Demyelination occurs broadly six weeks after cuprizone consumption and is called “acute demyelination”. When a cuprizone diet replaces regular chow, acute demyelination is followed by spontaneous remyelination [5].

Many types of organic and manufactured neuroprotective substances have been described. Synthetic neuroprotective drugs, however, are thought to have side effects, including fatigue, drowsiness, numbness in the upper and lower limbs, balance issues, nervousness, anxiety, etc. Scientists have, therefore, focused a lot of research on developing safe and effective neuroprotective medicines [1,6]. Adaptogens are plant-based medical and nutritional supplements that improve living organisms’ adaptation, resilience, and survival without harming the body’s homeostasis [4]. In this regard, seaweed may be a source of neuroprotective adaptogens [1].

Marine-derived natural substances have shown promise in exhibiting a range of pharmacological effects such as anti-diabetic, anti-inflammatory, antioxidant, anti-cancer, and anti-obesity actions. These findings suggest that such substances could be valuable for developing novel medications [7,8]. The valuable red macroalga *Palmaria p.* (Palmariales, Rhodophyta), often known as dulse, has been widely disregarded [9]. Rhodophyta, commonly known as red seaweed, is a large macroalgae group comprising approximately 7000 species. Seaweed contains many structurally diverse bioactive compounds, including protein, sulfated polysaccharides, pigments, polyunsaturated fatty acids, vitamins, minerals, and phenolic compounds. These substances have a range of applications in nutrition, medicine, and industry. Polysaccharides, representing 40–50% of the dry weight of red algal cell walls, are particularly valuable due to their ability to thicken and gel, making them useful in various industries and medicinal compounds [10].

Degenerative illnesses caused by inadequate fatty acid intake are among the leading causes of death for two thirds of people in wealthy, developed countries. The gut–brain axis controls degenerative diseases and obesity response. Western lifestyles promote peripheral inflammation and neuroinflammation with unhealthy diets. Through regular probiotic and dietary fiber ingestion, dysbiotic rehabilitation decreases obesity, mental disease, and gut–brain axis modulation [11,12]. Obesity-induced changes in food intake are associated with increased Firmicutes and decreased Bacteroidetes. Healthy fats (polyunsaturated omega-3 and omega-6 fatty acids) reduce cardiovascular disease, depression, and cognitive decline [13]. Linoleic acid (C18:2, n-6) and -linolenic acid (C18:3, n-3) are two polyunsaturated fatty acid )PUFAs( that humans and other vertebrates cannot produce [14]. The body of human beings easily assimilates *Palmaria p.* lipids. It contains a high concentration of eicosapentaenoic acid (EPA), a golden fatty acid (FA), in healthy diets that plays a role in preventing the development of non-communicable diseases, which contributes to 71% of all annual deaths worldwide. Eicosapentaenoic acid is an omega-3 (n-3) polyunsaturated fatty acid (PUFA) that improves and prevents cardiovascular and neurological disorders, as well as having antioxidant and anti-inflammatory properties [9]. In mice, n-3 PUFAs increased epithelial barrier function and reduced clinical colitis and intestinal immunopathology [9]. Clinical investigations indicated that n-3 PUFAs reduced Firmicutes/Bacteroidetes ratio, Coprococcus, Facecalibacterium, butyrate-producing Bifidobacterium, Lachnospira, Roseburia, and Lactobacillus [9,15,16,17]. In addition, a diet supplemented with n-3 PUFAs prevented neuropsychiatric disorders and dysbiosis caused by social instability stress during adolescence and maintained these effects into adulthood [18,19], supporting the idea that a healthy diet may prevent neurodegenerative disorders [20].

Protein hydrolysates of *Palmaria p.* have the potential to be multifunctional dietary components. Because of its antioxidant and tyrosinase inhibitory properties, it may have possible applications as a health-improving component and as a food preservative [21]. Phycobiliproteins (phycobilin at 100 or 500 g/mL) and chlorophyll “a” at 245 g/mL from *Palmaria p.* displayed anti-inflammatory action in lipopolysaccharides-stimulated murine macrophages (RAW 264.7 cells) by decreasing inflammatory mediators interleukin-6 (IL-6), tumor necrosis factor-alpha (TNF-α), and nitric oxide (NO) [22]. The safety of naturally-based medicines and mechanistic evidence of their usefulness in the cognitive domain still need to be better understood [6].

It is widely known that the gut microbiota (GM) is crucial in regulating the host’s physiology. Its composition and abundance can vary based on several factors, and it is composed of four main phyla (Bacteroidetes, Firmicutes, Proteobacteria, and Actinobacteria) and two minor species (Verrucomicrobia and Fusobacteria) [23]. *Palmaria p.* ingestion protected C57BL/6J male mice against obesity-related metabolic problems by boosting lipid excretion, decreasing systemic inflammatory markers, and moderating gut microbiota modification [24].

Davani-Davari reported that “a non-digestible food ingredient that beneficially affects the host by selectively stimulating the growth and/or activity of one or a limited number of bacteria in the colon, and thus improves host health” was the definition of prebiotic. This definition was established in 1995 and revised in 2008 to read as follows: “a selectively fermented ingredient that results in specific changes in the composition and/or activity of the gastrointestinal microbiota, thus conferring benefit(s) upon host health” [25]. *Palmaria p.* contains xylan, a xylose carbohydrate. Xylan has been demonstrated to regulate GM activity by increasing short-chain fatty acid synthesis with higher acetate, propionate, and butyrate levels. According to the latter, xylan from *Palmaria p.* is a fermentable fiber with putative prebiotic properties [26].

Increasing scientific research suggests that gut bacteria heavily influence the gut–brain axis. Given the reciprocal nature of this association, alterations in the composition of gut microbiota can affect neurological functions, and conversely, neurological changes may impact gut bacteria [23,27]. Intestinal mucosal barrier function (MBF) refers to the intestine’s ability to contain luminal bacteria and chemicals while facilitating nutrient absorption effectively. Red seaweeds and their constituents have demonstrated potential as functional foods that can enhance various components of MBF through their prebiotic, antioxidant, and immunomodulatory properties [28].

Most existing therapies can delay, but not cease, the worsening clinical progression. Modern medicines broadly impact the immune system, with the potential for substantial side effects. There is a possibility for prevention from MS, especially for most vulnerable persons, such as families of people with MS. As a result, effective MS therapies remain a medical need that is still unfulfilled [29]. Therefore, searching for naturally occurring neuroprotective agents could benefit those at risk for developing MS.

The primary objective of this study was to investigate, for the first time, the neuroprotective potential of *Palmaria p.* against multiple sclerosis, along with its probable impact on the gut microbiota during the disease course. Given antioxidant and anti-inflammatory properties, we assumed that *Palmaria p.* bioactive constituents could protect the brain against the cuprizone demyelinating effect and additionally can beneficially modulate the gut microbiota. Moreover, we postulated that there may be gender differences at the general morphology, behavioral, and microbiome levels. 

## 2. Results

### 2.1. Weight Assessment

Weight assessment was done weekly to assess any signs of toxic effects of CPZ or *Palmaria p.* on the weight and determine the suitable dose for each mouse. One-way ANOVA revealed insignificant changes among groups throughout the experiment (*p* > 0.05).

### 2.2. Palmaria p. Extract Fatty Acid and Antioxidant Assessment

Assessment of *Palmaria p.* fatty acids content revealed the presence of omega-3 (ω-3): eicosapentaenoic acid (EPA) (1.26 mg/mL), Docosahexaenoic acid (DHA) (0.584 mg/mL), and omega-6 (ω-6) Linoleic acid Methyl ester (1.669 mg/mL).

The ability of antioxidants to scavenge 2,2′-Azinobis (3-ethylbenzothiazoline-6-sulfonic acid) produced in the aqueous phase was measured using the Trolox Equivalent Activity Capacity (TEAC). At a TE of 3.94 ± 0.014 Trolox M TE/mg sample, the extract provided 60.4% inhibition of the ABTS radicals.

### 2.3. Behavioral Testing Results

#### 2.3.1. Grip Strength at W5 and W7 in Both Sexes

**Figure 1:**
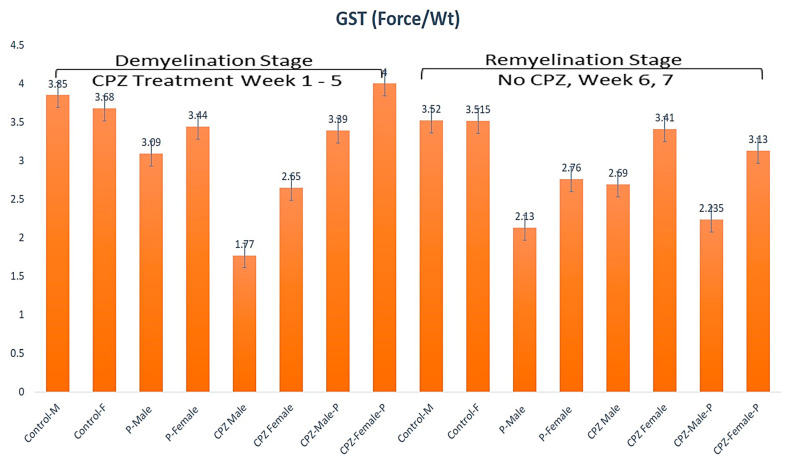
The grip strength among groups in weeks 5 and week 7.

In the current study, GS considerably reduced in both male and female groups treated with CPZ contrasted to the control group (*p* < 0.05), indicating accomplishment of the MS model (mean ± SEM as follows; control male: 3.85 ± 0.53, control female: 3.68 ± 0.13, CPZ male: 1.77 ± 0.12; CPZ female: 2.65 ± 0.15; *p* < 0.05) (Figure 1)**.** Analysis of the GST at the 5th week by two-way ANOVA revealed significant statistical differences among groups regarding gender and the type of therapy (*p* < 0.05, df = 1, F = 8.61, *p* < 0.1, df = 3, F = 25.1; respectively). The same trend was found at the end of the 7th week (*p* < 0.01, df = 1, F = 25.75, *p* < 0.1, df = 3, F =17.92, respectively). After CPZ withdrawal at the end of the remyelination stage, the CPZ + *Palmaria p.* groups recovered better than the CPZ-only groups. Females had better GS recovery than males in the CPZ + *Palmaria p.* groups. These findings are depicted in Figure 1.

#### 2.3.2. Open Field Test at W5 & W7 in Both Sexes

**Figure 2:**
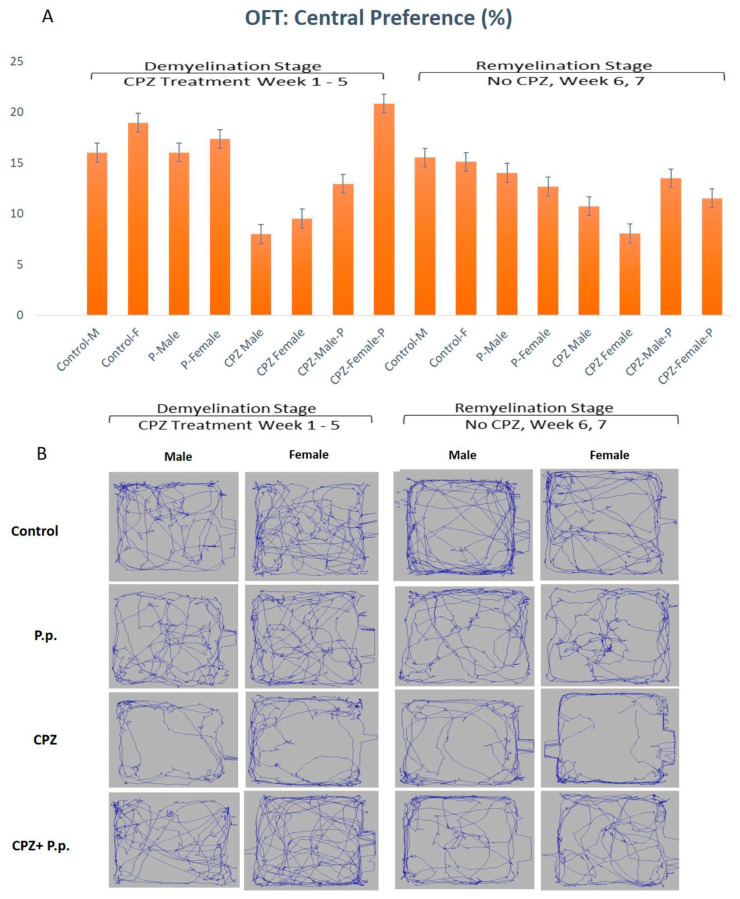
Open field test analysis in both the demyelination and remyelination stages. (**A**) shows the central preference of animals is represented in the percentage of the total time spent in the arena. (**B**) A diagram containing an example from each group representing the movement of the animal in the open field.

OFT-assessed parameters were the central preference, immobility, distance traveled, and velocity at the 5th and 7th weeks. The central preference in the 5th week displayed a statistically significant difference in gender and type of intervention among groups (*p* < 0.05, df = 1, F = 7.51 and *p* < 0.01, df = 3, F = 14.61, respectively). At the end of the 7th week, there were no statistically significant differences among groups regarding gender. The type of therapy showed a statistically significant difference (*p* > 0.05, df = 1, F = 0.95 and *p* < 0.01, df = 3, F = 2.96, respectively) (Figure 2). At the demyelination stage, the CPZ groups showed a diminished central preference contrasted to the control. The CPZ + *Palmaria p.* groups showed increased central preference toward normal compared to the CPZ groups, where females preferred the center compared to males (Figure 2).

**Figure 3:**
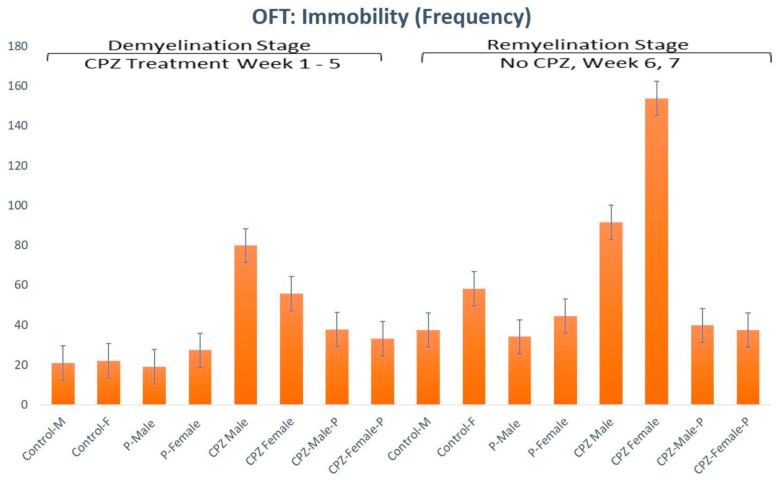
Open field test analysis for immobility frequency of animals in both the demyelination and remyelination stages.

Assessment of the immobility in the 5th week showed a statistically significant difference regarding gender and type of therapy given (*p* < 0.05, df = 1, F = 9.58 and *p* < 0.01, df = 3, F = 26.74, respectively). In the 7th week of the experiment, the immobility showed no statistically significant difference regarding the gender; however, the type of therapy was still significantly different (*p* > 0.05, df = 1, F = 2.0 and *p* < 0.01, df = 3, F = 9.28, respectively) (Figure 3).

Regarding the total distance traveled, there was no statistical gender difference. In contrast, the groups showed significant differences regarding the type of given intervention (*p* > 0.05, df = 1, F = 0.040 and *p* < 0.05, df = 3, F = 3.37, respectively). On the other hand, the same parameter revealed no significant statistical difference regarding the gender and the type of therapy in the 7th week (*p* > 0.05, df = 1, F = 1.07 and *p* > 0.05, df = 3, F = 0.89; respectively); Figure S1 in the Supplementary Materials.

Concerning the velocity in OFT in the 5th week, there was no statistically significant difference regarding gender. At the same time, the type of intervention revealed substantial differences among groups (*p* > 0.05, df = 1, F = 0.00 and *p* < 0.05, df = 3, F = 3.71, respectively). In comparison, the velocity revealed no statistically significant difference in the 7th week concerning both gender and the type of therapy (*p* > 0.05, df = 1, F = 0.88 and *p* > 0.05, df = 3, F = 1.21, respectively); Figure S1 in the Supplementary Materials.

### 2.4. Gut Microbiota Analysis

#### 2.4.1. Diversity in the Gut Microbiome Community

The study analyzed 3,264,873 high-quality reads from fecal samples of various mice using metagenomics amplicon. The sequences were grouped into 3039 operational taxonomic units (OTUs), with Bacteroidetes, Firmicutes, and Proteobacteria comprising over 97% of the phyla in each sample, except the male control group. Other species detected include Actinobacteria, Deferribacteres, Tenericutes, and others. The study found that most bacterial content in the samples had a Good Coverage of 99% or more, Table 1 and Figure S2.

The study used principal coordinate analysis to measure beta diversity, which indicates the similarity or dissimilarity between samples. The analysis showed that gut microbes from certain groups of mice clustered together. The GM from demyelinated animals clustered differently than those from the remyelinated stage.

Alpha diversity is a measure of diversity in a single sample and can be estimated through various methods, such as rarefaction analysis, the Chao1 index, and the Shannon index. The Chao1 index forecasts the total number of species in each sample, while the Shannon index estimates the adequate number of species, Table 1.

The Palmaria male group had the highest Chao1 value of 281.04, followed by the male control group with a Chao1 index of 250.05. As indicated by the Shannon index, the lowest evenness was observed in the CPZ-male-*Palmaria* group and the male control group sacrificed in the 7th week, with values of 3.14 and 3.15, respectively. The highest evenness was found in the male control group in the 5th week and *Palmaria p.* female group in the 5th week. The CPZ-male-*Palmaria p.* group sacrificed in the 7th week had the lowest diversity (67.57%) on Simpson’s Diversity Index. This score considers the number of species and their relative abundance, with higher richness and evenness suggesting more variety, Table 1.

#### 2.4.2. Variations in GM in Both Sexes at the End of Demyelination and Remyelination Stages

Our data shows that male mice had fewer Bacteroidetes than female mice at the end of the 5th week of age (28% vs. 40%, respectively), but no gender difference was observed in firmicutes and proteobacteria populations. The administration of CPZ resulted in an expansion in the Bacteroidetes community and a decline in Proteobacteria in both sexes. However, the administration of *Palmaria p.* along with CPZ to male mice resulted in a decrease in Bacteroidetes (40.5%) and a rise in Firmicutes (51.2%) compared to the CPZ-male mice group (57.6% and 34.6%; respectively). *Palmaria p.* did not affect the population of Firmicutes in female mice treated with CPZ, but it did increase the population of Firmicutes at five weeks when administered alone. No changes were observed in the population of Bacteroidetes when mice were given *Palmaria* alone compared to control mice, Table 2.

**Figure 4:**
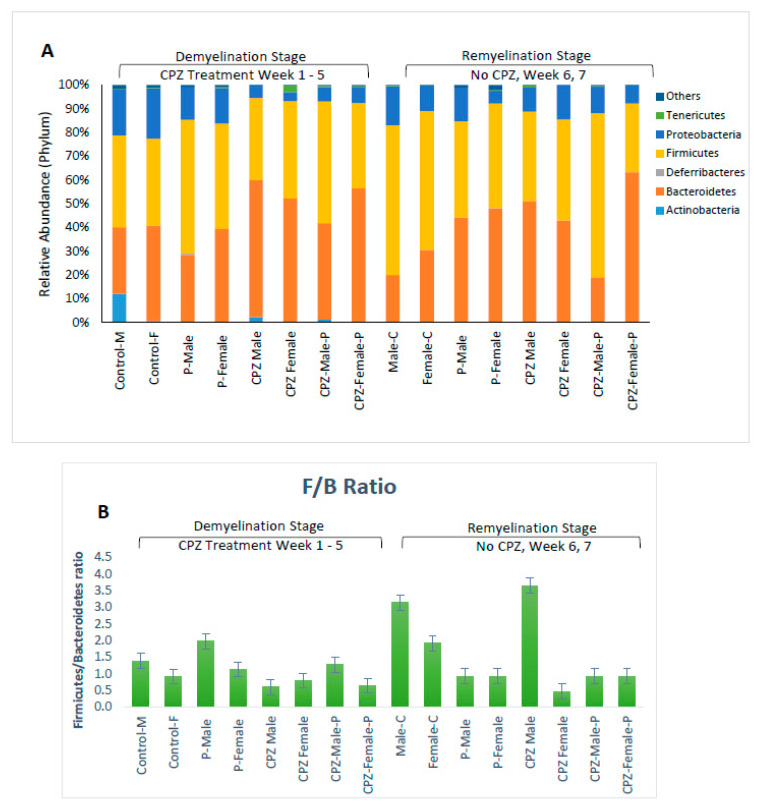
Effects of Cuprizone and Palmaria palmata on the composition of the gut microbiota at the phylum level. Relative abundance at the phylum level (**A**), Distribution of microbiome at the phylum level (**B**), Firmicutes/Bacteriodetes ratio.

**Figure 5:**
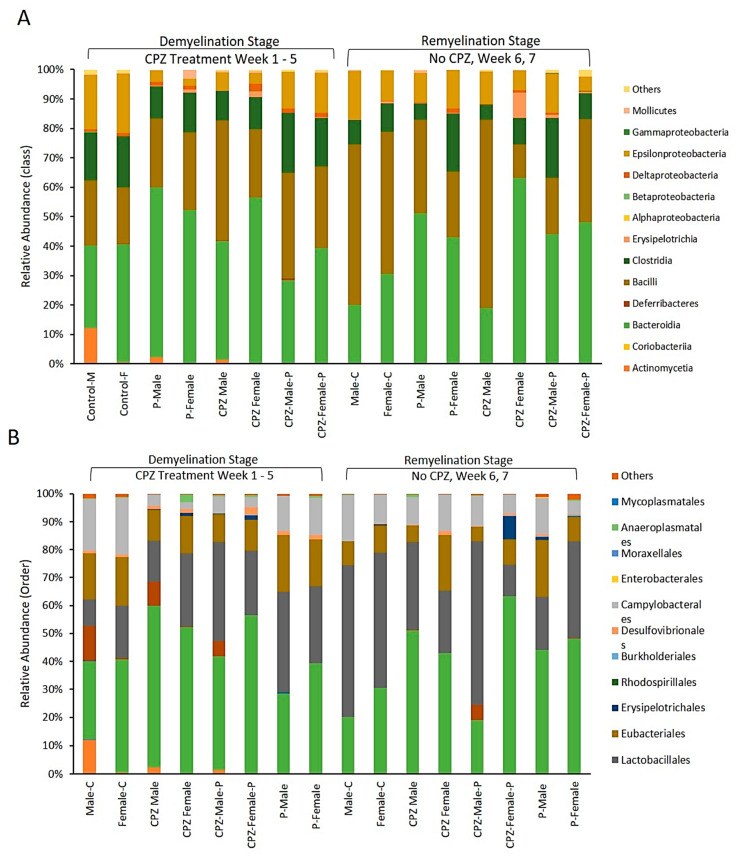
(**A**): Effects of Cuprizone and Palmaria palmata on the composition of the gut microbiota at the class level, showing the relative abundance of the microbiome. (**B**): Effects of Cuprizone and Palmaria palmata on the composition of the gut microbiota at the Order level showing the microbiome relative abundance.

**Figure 6:**
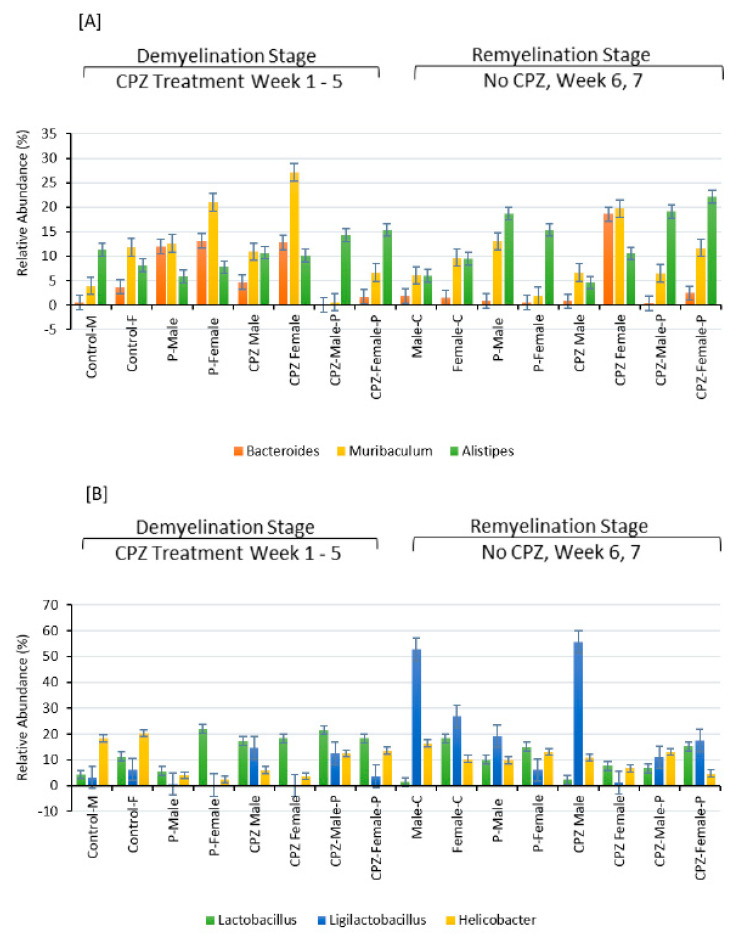
Effects of Cuprizone and *Palmaria palmata* on the composition of the gut microbiota at the Genus level. Relative abundance at the genus level, relative levels of different genera in (**A**,**B**).

At the remyelination at the end of the 7th week, mice that received CPZ showed a rise in Bacteroidetes and a diminishment in Firmicutes compared to the control group. However, female mice that received CPZ and *Palmaria p.* showed a significant expansion in Bacteroidetes and reduction in Firmicutes communities, which was not observed in male mice. These results indicate that the gut bacterial community is influenced by factors such as CPZ, *Palmaria p.*, age, and gender, as illustrated in Figure 4, Figure 5 and Figure 6.

The Firmicutes to Bacteroidetes (F/B) ratio:

In male mice, the F/B ratio increased significantly when CPZ was stopped with continued *Palmaria p.* supplementation, almost exceeding the control level. In female mice, the stoppage of CPZ in the CPZ + *Palmaria p.* group led to a decrease in the F/B ratio at the end of the 7th week compared to the end of the 5th week. These data imply that both CPZ and *Palmaria p.* may influence the gut microbiota and the F/B ratio and that the effects may vary depending on the gender of the mice. Additional research is required to completely comprehend the processes driving these changes and their possible health consequences, Figure 5.

Fourteen classes were identified in the gut of the studied animals, with four categories being dominant. The abundance of the Actinomycetia class was 12.1% in the male-control group at the end of the demyelination stage, but it disappeared entirely as the animals aged to 7 weeks. In contrast, the Actinomycetes population was deficient in the female-control group and other groups at corresponding time points. Administration of CPZ was found to decrease the population of Epsilonproteobacteria, but interestingly, their population was restored upon withdrawal of CPZ from the diet, Figure 6A.

Concerning the order of microbiota, CPZ treatment led to an expansion of Bacteroidales and Lactobacillales populations in both male and female mice. However, it decreased the populations of Eubacteriales and Campylobacteriales compared to their respective control groups, as shown in Figure 6B. Regarding the genus of microbiota, the combined use of *Palmaria p.* and CPZ resulted in an increased abundance of Lactobacillus and Ligilactobacillus in the male group and Murabiculum in the female group, as depicted in Figure 6.

### 2.5. Histopathological Assessment of the Corpus Callosum in Demyelination and Remyelination Stages in Both Sexes

**Figure 7:**
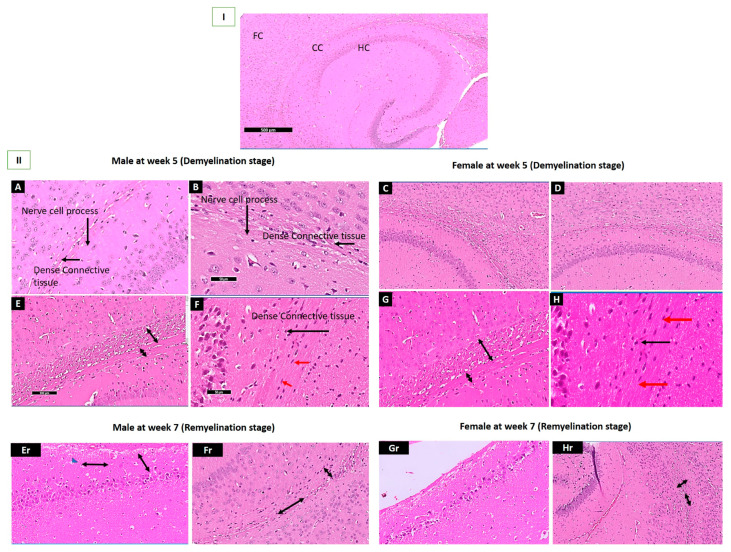
**I**: A section revealing the corpus callosum, hippocampus, and frontal cortex. (**II**-**A**,**C**); A photomicrograph of Sections of the control group showing the typical architecture of the brain of adult mice with the apparent typical architecture of the Corpus Callosum (CC) at the end of the 5th week. Notice the presence of nerve cell processes within dense connective tissue fibers (black arrows). (**II**-**B**,**D**); A photomicrograph of Sections *Palmaria p.* of mice brain showing the typical architecture in both male and female mice with the apparent typical architecture of Corpus Callosum (CC) at the end of 5th week. Notice the presence of nerve cell process within between dense connective tissue fibers. (**II**-**E**,**G**); A photomicrograph of a section in the brain of CPZ groups at the end of the 5th week of male and female mice showing apparent thinking of the CC and increased dense connective fibers. (**II**-**F**,**H**); A photomicrograph of Sections of the CPZ + *Palmaria p*. groups at the end of the 5th week of adult male and female mice showing thickening of CC with an increase of dense connective (black arrow), evidence of typical architecture of Oligodendrocytes (red arrow). (**II**-**Er**,**Gr**); A photomicrograph of a section in the brain of male and female mice at the end of the 7th week shows apparent thinking of the CC and an increase in the dense connective fibers. Notice the marked decrease in the vacuolation with a reduction in the cellular infiltration in males (blue head arrow). At the same time, there is a mild increase in cellular infiltration in females. (**II**-**Fr**,**Hr**); A photomicrograph of a section in the brain of adult male and female mice of the CPZ + *Palmaria p.* groups at the end of the 7th week showing apparent mild thinking of the corpus callosum, as well as increased dense connective fibers and no vacuolated cytoplasm as well as a decrease in the cellular infiltration. H & E, × 200.

Figure 7I displays the standard architecture of the HC, CC, and FC. The *Palmaria p.* group of both sexes revealed myelinated nerves, normally distributed Schwann cells, and nerve cell processes between dense connective tissue fibers, indicating no abnormalities; Figure 7(II-A,C). However, in the affected group (CPZ) at the end of the fifth week for both sexes, revealed a thickening of the CC and dense connective fibers, along with marked vacuolated cytoplasm, cellular infiltration in the nerve cell, pyknotic nuclei, and demyelinated nerves Figure 7(II-E,G) and Figure S3A,B. The CPZ + *Palmaria p.* at the demyelination stage section reveals the standard architecture of the CC, and myelinated nerves maintain typical architecture; Figure 7(II-F,H).

At the remyelination stage, there is a marked decrease in vacuolation, and a reduction of cellular infiltration is observed in males. In contrast, a mild increase in cellular infiltration is seen in females in the CPZ group, as shown in Figure 7(II-Er,Gr). At the same time, CPZ + *Palmaria p.* groups of adult male and female mice at the end of the 7th week revealed a mild thickening of the corpus callosum, increased dense connective fibers, and no vacuolated cytoplasm, in addition to a decrease in cellular infiltration, Figure 7(II-Fr,Hr) and Figure S2A,B. The vacuolation of the cytoplasm appears to be less pronounced in the male sections compared to the female sections, Figure S4 in the Supplementary Materials.

## 3. Discussion

The study examined the neuroprotective effects of red seaweed *Palmaria p.* on CPZ-induced MS in a mouse model, concentrating on the role of gender and the variance of gut microbiota. Results showed that administration of *Palmaria p.* in combination with CPZ resulted in positive changes in structural, behavioral, and gut microbiome communities. Both sexes showed improved structural changes, as revealed by CC. This is the first study to assess the role of gender in the neuroprotective effects of *Palmaria p.* on MS. The study found that females recovered more slowly than males after the cessation of CPZ in the CPZ + *Palmaria p.* group. The GST showed increased strength in both sexes, with female predominance during the demyelination stage only. In the OFT, CPZ + *Palmaria p.* showed decreased anxiety-like behavior contrasted with the CPZ group during the demyelination stage, with a gender difference being obvious regarding central preference and immobility. The study team observed that combining *Palmaria p.* with CPZ caused alterations in the gut microbiome, including a decline in the F/B ratio and a rise in the relative abundance of Lactobacillus and Bacteroidia populations, both of which have been related to favorable effects on the host. These changes showed sex differences. *Palmaria p.* contained ω-3, a neuroprotective agent that positively affects the gut microbiome and has an antioxidant capacity, which could contribute to its neuroprotective effect.

### 3.1. The Neuroprotective Effect of the Palmaria p. Bioactive Constituents on the Structure and Function

Polyunsaturated fatty acids (PUFAs), omega-6, and omega-3 are essential fatty acids that humans or other mammalian species cannot produce due to a lack of naturally occurring enzymes for omega-3 desaturation. The presence of ω-3 fatty acids (EPA & DHA) and -6 (Linoleic acid) was identified in the current study’s *Palmaria p.* fatty acids analysis. Foseid et al. documented *Palmaria p.* had a high concentration of ω-3 PUFA (19.0 ± 0.7%) but had a lower overall ω-6 content (1.3 ± 0.1%). The ω-6/ω-3 ratio was ≤0.3 [30]. There is a vital genetic component in determining PUFA content based on the consistency of several types of research on PUFA content between macroalgal species. The quantity of each species and the impact of the environment on PUFA content and composition are currently being debated [14]. One of the critical pathophysiological processes of neuropsychiatric and neurodegenerative illnesses is inflammation. Despite the relevance of inflammation in many diseases, anti-inflammatory therapies are lacking. Omega-3 polyunsaturated fatty acids (n-3 PUFAs) can inhibit inflammation by producing specialized pro-resolving mediators (SPMs) like resolvins D (RvD) and E (RvE) series, maresins (MaR) and protectins (PD) [31,32]. SPMs from EPA and DHA have comparable but distinct effects on cell death pathways and anti-inflammatory and post-inflammatory resolution [32]. Dietary or de novo-generated EPA and DHA reside in the lipid bilayer and play a critical role in cellular processes. EPA and DHA exhibit various membrane contacts (molecular positions and orientations) in the lipid bilayer, affecting signal transmission, fluidity, lipid oxidation, and cholesterol domain formation [32]. The findings of a meta-analysis conducted on cohort studies suggest an inverse association between blood ω-3 fatty acids concentrations and the expression of inflammatory genes. This observation implies that blood ω-3 fatty acid concentrations might serve as promising indicators for the diagnosis, prognosis, and therapy of MS [33]. However, more clinical studies are necessary to validate the possible impact of ω-3 fatty acids on multiple sclerosis therapy.

Antioxidants can neutralize the radical cation ABTS • + either directly through electron donation or indirectly through hydrogen atom donation [34]. Harnedy et al. discovered a novel decapeptide with antioxidant properties from *Palmaria palmata* aqueous extract. The ferric-reducing antioxidant power (FRAP) activities of this decapeptide were 21.23 ± 0.90 nmol Trolox equivalents (TE)/mol peptide [21,22]. In macroalgae like *Palmaria p.*, epicatechin is the most prevalent phenolic component. One of this chemical’s most essential biological actions is its ability to scavenge reactive oxygen species (ROS). Epicatechin reduced ROS levels while increasing GSH concentration. Another study found that epicatechin had anti-inflammatory characteristics by inhibiting TNF-, iNOS, and NF-B expression increases in a doxorubicin-treated rat model [22]. Polyunsaturated fatty acids (PUFAs) modulate antioxidant signaling pathways by incorporating them into cellular membranes. The high docosahexaenoic acid (DHA) concentration in eukaryotic mitochondrial membranes shows that DHA is an essential phospholipid in oxidative phosphorylation, which generates ATP. DHA reduces oxidative stress and cytochrome c oxidase (complex IV) activity in the mitochondria while increasing manganese-dependent superoxide dismutase (Mn-SOD) activity. A fish oil-rich diet boosted superoxide dismutase expression and activity in rats. Thio-barbituric acid (TBARS) products decreased in these rats, indicating membrane peroxidation reduction [35,36]. Both human and rat studies have demonstrated that EPA and DHA may lower urine F2-isoprostane levels, a marker for oxidative stress [35]. In the current research, we found that *Palmaria p.* aqueous solution has an antioxidant capacity that leads to inhibition of the free radical activity as assessed by TEAC by 60.4%. This concurs with previous studies investigating *Palmaria p.* extract [37,38].

Notably, the neuroprotective role of *Palmaria p.* in the CPZ-induced mice model of MS was noticed in the current study by preserving the neural architecture of CC in the groups treated with *Palmaria p.* compared to the CPZ-treated groups. The CPZ model’s first clinical observation was that the severity of symptoms and mortality rate were related to CPZ dose and treatment duration [39]. Demyelination of the CC in the CPZ model has been linked to impaired motor coordination between the left and right paws [40]. Earlier reports documented that the age of the mice also played a role, as weanling mice experienced significant growth reduction and weight loss after CPZ treatment [41].

In the current study, it has been observed that CPZ, during the demyelination stage, led to diminished GS, which has been improved with treatment by *Palmaria p.*, indicating the potential protective effect of the latter. Notably, the gender difference was prominent during the remyelination stage more than the demyelination stage, with males being more affected by CPZ and showing lesser recovery than females during the remyelination phase. Regarding the sex differences, it has been reported in human studies that although women have a more robust immune response associated with MS, men often have a more advanced neurodegenerative pathology and prognosis [42]. Women have an earlier onset of MS and more relapses in relapsing-remitting (RRMS) types. Still, males acquire impairment quicker, attain disability milestones sooner, and recover more slowly after the initial illness relapse [43,44]. Therefore, hormonal or genetic variables are involved in controlling disease progression, and sex hormones, including estrogens, progesterone, prolactin, and androgens, are likely to play a part in these complicated pathways [44]. This could explain our study results related to better recovery in females after the withdrawal of CPZ.

Both central preference and immobility can assess the emotionality of the mice. The animals are close to the arena’s walls when they feel anxiety, called “thigmotaxis” [45]. In the current study, the OFT revealed that CPZ decreased the central preference and immobility in the demyelination stage compared to the control group. This decrease was prevented in the group treated with *Palmaria p.* Therefore, it is apparent that CPZ leads to increased anxiety levels in animals, and *Palmaria p.* has protected from this effect in the treated groups. 

### 3.2. Palmaria p. Affected Gut Microbiome Community Variably Regarding the Disease Stage and the Sex

The Firmicutes are involved in gut catabolic reactions and inhibit pathogenic bacteria via their protective actions against intestinal inflammation [46]. An earlier study on humans revealed that Firmicute abundance was low in MS patients [47]. Some studies found increased firmicutes in MS patients, while others found a decline. Firmicutes, which can cause inflammation, include Streptococcaceae, Ruminococcaceae, and Lachnospiraceae. On the opposing side, MS patients frequently had lower Bacteroidetes, Prevotellaceae, and Bacteroidaceae levels. MS patients had higher concentrations of actinobacteria, such as Bifidobacteriaceae, and proteobacteria, such as Desulfovibrionaceae [48].

In the current study, the relative abundance of the genus Alistipes (Phylum Bacteroidetes) was increased when *Palmaria p.* was given to the mice. It has been reported that dysbiosis in the Alistipes population may affect the host positively or negatively. Flavonoids from red seaweed Enteromorpha prolifera have been reported to increase the abundance of Alistipes, Lachnospiraceae, and Odoribacter genera in diabetic mice [49].

Proteobacteria include Escherichia coli, Salmonella, and Campylobacter, which are opportunistic pathogens [50]. In the present study, we noticed a decrease in the Proteobacteria population with CPZ treatment. This decline was diminished in the groups treated with a combination of CPZ+ *Palmaria p.* Nevertheless, the administration of *Palmaria p.* alone decreased the Proteobacteria abundance to a lesser level. Proteobacteria comprise a small percentage of the normal gut microbiota, but if their numbers rise out of proportion, it could lead to generalized inflammation [51]. Previous reports documented some Proteobacteria Phylum abundance types seen in MS patients. However, a few trials have shown that after MS patients received medications to control their condition, Sutterella (a variety of proteobacteria) levels returned to normal. These findings suggest that Proteobacteria play a part in both pro- and anti-inflammatory processes in MS [52]. Therefore, *Palmaria p.* may regulate the abundance of this Phyla toward the beneficial direction. However, this assumption needs to be confirmed by further investigations.

Despite significant accomplishments, more needs to be understood about how the microbiome affects the host and vice versa. The absence of standardized study procedures, sampling techniques, or ribosomal gene sequencing strategies could all be technical causes for this. Other potential influences include environmental elements that affect the gut microbiota, such as food and weather [48,53,54].

### 3.3. The Suggested Role of Palmaria p. Active Constituents in Modulating Gut–Brain Axis

Per the latest studies, *Palmaria p.* can be exploited as a source of EPA-rich lipids, the antioxidant potential for food and feed, and nutraceuticals [5]. The human body readily absorbs *Palmaria p.* lipids and includes a high concentration of eicosapentaenoic acid (EPA), a golden fatty acid (FA) found in healthy diets [9]. 

Dietary omega-3 polyunsaturated fatty acids (PUFAs) have been connected to the modulation of gut immunity and maintaining gut homeostasis. Omega-3 PUFAs influence the gut microbiota in three distinct manners: (1) by influencing the diversity and quantity of gut microorganisms; (2) by altering the quantities of proinflammatory cytokines such as endotoxins (lipopolysaccharides) and IL17; and (3) by influencing the quantities of short-chain fatty acids (SCFAs) or short-chain fatty acid salts [55,56].

Increasing the number of anti-inflammatory substances like SCFAs and omega-3 PUFAs can also impact gut microbial flora [57]. In a case study evaluating the influence of an omega-3 PUFA-rich food on the human gut microbiome, an increase in numerous SCFA (butyrate)-producing taxa, including Bacterioides, was observed [58,59]. Additionally, the microbiota stimulates immunological and neuronal cells in the brain and enhances brain functions [23]. In the current study, *Palmaria p.* combination with CPZ prevented the decrease in the F/B ratio. Dietary omega-3 PUFAs can lessen the decline in the F/B ratio in mice with a high-fat diet [60]. This is in line with our results in the current study.

PUFAs are necessary for the growth and function of nerve tissue. Performance problems in cognition and behavior are linked to low PUFA levels. GM has been linked to the reduction of stress and anxiety. For example, Lactobacillus rhamnosus, as a probiotic, has been identified as an anxiolytic [61,62]. An earlier study on humans with test anxiety revealed that test anxiety and probiotics had little effect on the abundance of Firmicutes and Bacteroidetes at the phylum level. At the same time, Actinobacteria could be significantly reduced, and probiotic intake could not restore it to normal levels [63]. There still needs to be more knowledge about the relationship between gut microbes and behavior. This could be attributed to the intermingling multifactorial effect on gut microbes like sex, age, species, and type of interventions.

Additionally, the gut–brain axis is bidirectional. Therefore, anxiety could affect the microbes and vice versa. Therefore, in-depth studies that address all these intervening factors are essential. 

Omega-3 fatty acids, EPA and DHA, are believed to have roles in neurogenesis, neurotransmission, and defense against oxidative stress. They achieve some of these effects by inhibiting the synthesis of proinflammatory prostaglandin E2 (PGE2) caused by omega-6 PUFA, such as arachidonic acid (AA) [64]. An earlier investigation found that all rat groups given EPA-supplemented diets experienced a delayed onset of clinical illness. During the acute period of EAE, this effect was linked to a rise in the expression of myelin proteins and an improvement in the structure of the myelin sheath [65].

**Figure 8:**
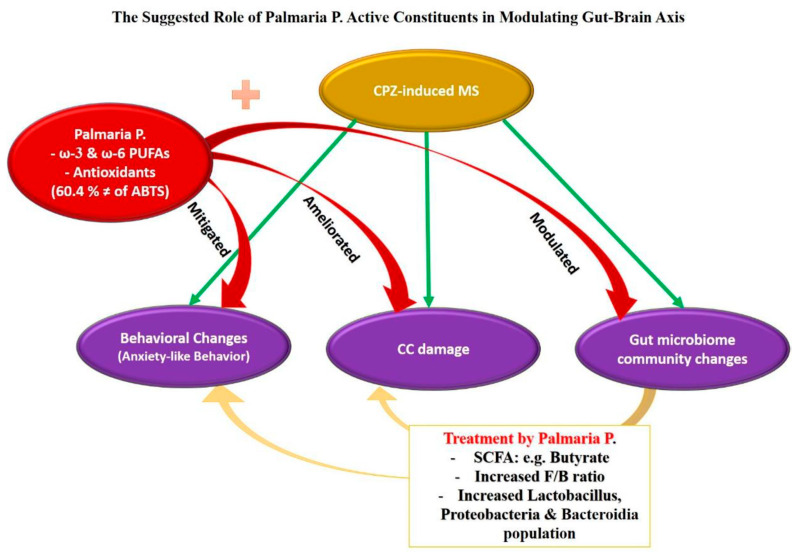
In the current study, CPZ induced behavioral changes in OFT through anxiety-like behavior. Additionally, it leads to damage in the corpus callosum (CC) and changes in the microbial community suggested previously as a predisposing factor for multiple sclerosis. When CPZ was combined with *Palmaria p.*, it mitigated the behavioral effect of CPZ, ameliorated the CC damage, and positively changed the gut microbiome community. Based on our results and previous studies, we argue that these beneficial effects of *Palmaria p.* could be related to its antioxidant properties and, more importantly, its possession of the healthy fatty acid omega-3 (ω-3). Moreover, omega-3 PUFAs could influence the gut microbiome by modifying the variety and abundance of gut microbes and by controlling the concentrations of short-chain fatty acids (SCFAs) such as butyrate. A rise in SCFA (butyrate)-producing genera is essential for supporting human gut health. The increased F/B ratio and an increase in the Lactobacillus, Proteobacteria, and Bactriodia communities after consumption of *Palmaria p*. in the current study are suggested to be the underlying neuroprotective factors against MS.

From the discussion, it can be concluded that there is a promising role of *Palmaria p.* in modulating the gut microbial community and mitigating neural effects produced in mouse animal models. This can be attributed to its rich content of PUFAs via various mechanisms connecting the gut to the brain. In addition, the antioxidant properties of *Palmaria p.* play a key role (Figure 8).

## 4. Methods

### 4.1. Animals

A total of 88 SWR Swiss mice were kept in a controlled environment with appropriate humidity, temperature, and a cycle of 12 h light and darkness, comprising 44 males and 44 females with an approximate weight range of 20–25 g and an age of 8 ± 2 weeks. Throughout the experiment, the mice had unlimited access to food and water. The experimental protocols complied with the ACUC committee’s guidelines and the animal house of FFMRC and received approval from the KAU biomedical ethics research committee (Reference No 368-21).

### 4.2. Chemicals

#### Cuprizone (CPZ) and *Palmaria p.*

Cuprizone (Thermo Fisher Scientific-C9012-25G; Waltham, MA, USA) was freshly prepared daily and blended with ground regular chow in 0.2% *w/w* of CPZ [66]. *Palmaria p.* leaf Powder Liquid Extract 2 × 4 oz was purchased from HawaiPharm LLC (Honolulu, HI, USA). The effect of CPZ and *Palmaria p.* on body weight was measured by the percentage of body weight gain throughout the seven weeks of the experiment following this equation: weight gain (%) = (new weight [W1] − initial weight [W0]/initial weight [W0]) × 100 [67].

### 4.3. Palmaria p. Extract Characterization

According to Arnao et al.’s approach, 192 mg of 2,2′-Azinobis (3-ethylbenzothiazoline-6-sulfonic acid) (ABTS) was dispersed in distilled water and placed in a 50 mL volumetric flask, then the volume was finished with distilled water [68], with slight adjustments to be carried out in microplates. Moreover, 1 mL of the previous solution was combined with 17 L of 140 mM potassium persulphate and incubated for 24 h in the dark. Diluting 1 mL of the reaction mixture to 50 mL with methanol yielded the final ABTS dilution utilized in the test. Moreover, 190 µL of The freshly produced ABTS reagent was mixed with 10 L of the sample/compound on a 96-well plate (*n* = 6), and the reaction underwent incubation at ambient temperature for 30 min in the dark. The decrease in 2,2′-Azinobis (3-ethylbenzothiazoline-6-sulfonic acid) (ABTS) at 734 nm [69] color intensity was observed at the end of the incubation time. The following Equation (1) is used to represent data as means SD:

(1) percentage inhibition = (average absorbance of blank − average absorbance of the test Average absorbance of blank) × 100

The FluoStar Omega microplate reader was used to record the results. Micro molar Trolox equivalent per mg sample (µM TE/ mg sample).

### 4.4. Experimental Design

**Figure 9:**
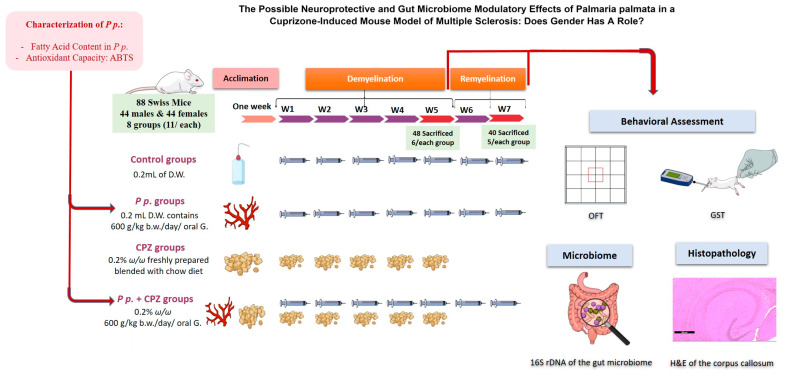
Assessment of the animal among groups throughout the experiment timeline from week one to week 7. A graphical abstract revealing the stages of the current study. To our knowledge, the study novelty encompasses the usage of the red marine algae, *Palmaria palmata*, in managing multiple sclerosis for the first time. Additionally, we investigated the effect of *Palmaria p*. as a prebiotic that can positively affect the gut microbe community and hence can lead to neuroprotection against multiple sclerosis. *Palmaria p*. was purchased and characterized for its fatty acid content and antioxidant capacity. The Swiss mice were left to acclimate for one week before the experiment. The experiment consisted of two stages: a demyelination stage and a remyelination stage. Eighty-eight mice (44 males and 44 females) were assigned randomly to the groups. Each gender was then subdivided into four main groups to pass through the two stages of the experiment. The groups were control groups, *Palmaria p*. groups, Cuprizone (CPZ) groups, and CPZ + *Palmaria p*. groups. The treatments were all given daily by oral gavage for 5 weeks. At the end of the 5th week, behavioral assessments were done, CPZ was stopped, and six animals from each group were sacrificed to assess the brain histopathology and the gut microbiome. The rest of the animals continued on the interventions, except for the withdrawn CPZ from all groups. At the end of the 7th week, behavioral assessments were repeated, and animals were then sacrificed for histopathology and microbiome assessments. *P.p.*: *Palmaria palmata*, D.W.: disitlled water, *w*/*w*: weight by weight, b.w.: body weight, oral G: oral gavage.

One week before the experiment began, the animals were kept for acclimatization. Animals were divided randomly into eight groups (4 males and 4 females; *n* = 11/each). The group was categorized as follows: Control groups (o.2 mL of distilled water), CPZ-fed groups (CPZ-mixed chow 0.2% CPZ (*w*/*w*) [66], CPZ + *Palmaria p.* groups (CPZ-mixed chow and 0.2 mL of 600 g/kg *Palmaria p.*) and *Palmaria p.* groups (600 g/kg *Palmaria p.*) for five weeks duration [70]. After five weeks, the CPZ was stopped in all groups, and *Palmaria p.* was continued without CPZ to assess its potential effect for better recovery. Six animals were sacrificed from each group by decapitation after thiopental anesthesia at the end of the 5th week to determine the microbial community at the demyelination stage of the disease. After another two weeks (at week 7), the rest of the rats in all groups were sacrificed to assess the microbial community in the remyelination stage. Histological assessment of the demyelination stage and remyelination was done to examine the effect of *Palmaria p.* on improving the pathological changes induced by CPZ. The colonic specimens from the middle part of the transverse colon were collected and processed for further microbiome analysis (Figure 9).

### 4.5. Behavioral Testing

#### 4.5.1. Grip Strength Test (GST)

The GS of the mice was measured using a computerized GS meter (Columbus Instruments, Model: 1027SM grip strength meter), consisting of a metal grid bar connected to a force transducer and digital display. To assess the forelimb GS, the investigator gently held the mouse by the base of its tail while allowing it to grasp the metal bar with its forepaws. The peak force of each measurement was automatically recorded in grams (g). Each mouse’s forelimb grip strength was assessed in triplicate [71] with about one-hour time intervals between the measurements.

#### 4.5.2. Open Field Test (OFT)

To assess the locomotor activity and the mood of the animals’ OFT parameters were used according to the previously published protocols [72]. At the start of the experiment, each mouse was positioned in an open-field arena (45 cm × 45 cm^2^) and allowed to roam freely for 3 min. A video camera placed above the box tracked and recorded the mice’s movements for further offline analysis of the data [67]. The total distance (cm) traveled and velocity (cm/s) of movement was used to assess the locomotor activity of the animals. While the central preference and immobility were used to determine the mood. All parameters were measured using the EthoVision tracking system [67].

### 4.6. Sequencing of Bacterial 16S rDNA Gene and Data Processing

Using the Quick-DNA Fecal/Soil Microbe Microprep kit, the entire genomic DNA from the animal’s intestines (mid-colon) was retrieved (Zymo Research, Irvine, CA, USA) using the nanodrop device to verify quality and quantity per the manufacturer’s instructions (ThermoScientific, Waltham, MA, USA). Targeting the V3 and V4 area with particular primers, the extracted DNA samples were submitted to 16S rDNA gene sequencing: 341F (50-CCTACGGGNGGCWGCAG-30) and 805R (50-GACTACHVGGGTATCTAATCC-30). The purified amplicons were submitted to sequencing on an Illumina MiSeq platform following PCR amplification and purification (Macrogen, Seoul, Republic of Korea). The sequences were combined into an operational taxonomic unit (OTU) with a 97% similarity after the Illumina MiSeq data analysis. QIIME (version 1.9) was used to examine the Chao1, Shannon, and ACE alpha diversity indices. The principal coordinate analysis (PCoA) plot was visualized using EMPEROR, and the beta diversity was calculated using the unweighted UniFrac distance metric.

### 4.7. Histological Assessment of the Brain Tissue

The brain tissue was dissected after the animals had been sacrificed. The specimens were dehydrated in ethyl alcohol for increasing strength for two days and then cleared in xylol. As customary, paraffin blocks were made, cut into five slices, and stained with hematoxylin and Eosin before being inspected under a light microscope [73]. The samples were preserved for ten days in 10% neutral formalin. They were dehydrated as the ethyl alcohol concentration increased (50%, 70%, 96%, and absolute alcohol). Xylol was used to clean the samples. Afterward, the tissues were coated with soft paraffin and repeatedly submerged in oven-melted wax with a melting point of 50 °C. Lastly, the tissues were dissolved in melted wax (melting point 55 °C), poured into a mold, and cooled to create paraffin blocks holding the tissues. Hematoxylin and Eosin were used to stain serial sections 5–7 um thick and put on plates [74]. All Sections were examined with an electrical microscope, and photographs were obtained using a digital pathology scanner in the Alborg pathology Lab.

### 4.8. Statistical Analysis

The study results are expressed as mean ± standard error of the mean (SEM). Statistical analysis was performed using IBM SPSS statistics 25, with one-way ANOVA followed by Dunnett’s multiple comparisons test or Kruskal–Wallis test followed by Dunn’s multiple comparisons test for microbiome analysis. For behavioral studies, two-way ANOVA was applied to assess the effect of grip strength and the OFT parameters (dependent variables) on the types of interventions and gender (independent variables). The degree of freedom (df) and F-value were reported. A P-value of less than 0.05 was considered statistically significant. Graphical abstracts were generated using Mind the Graph and Microsoft PowerPoint tools.

## 5. Limitations and Conclusions

Although the study did not establish a specific pathway linking the gut microbiome and the brain, it was the first to examine the beneficial impact of *Palmaria p.* on male and female mice with MS. The study’s objective was to investigate the potential behavioral and microbial changes that could arise by considering the mechanisms described in previous research. Another limitation is that we assessed only the preventive role of *Palmaria p.* against a chemically induced model of MS. Therefore, further studies evaluating the therapeutic effect of *Palmaria p.* with other models of MS are still needed.

Collectively, the study found that *Palmaria p.* effectively mitigates with variable degrees of CPZ-induced MS in both male and female Swiss mice. This was demonstrated by *Palmaria p.*, which can potentially protect against CPZ-induced MS with varying degrees in male and female Swiss mice. This protection was demonstrated through three key findings: (1) increased F/B ratio and expansion of the beneficial Lactobacillus, Proteobacteria, and Bactriodia communities. (2) Protection against the decline in GS induced by CPZ and prevented CPZ-induced anxiety in OFT. (3) Preservation of structural integrity. Therefore, *Palmaria p.* could be a potential probiotic for the at-risk groups as a preventive measure against MS.

## Figures and Tables

**Table 1:** Alpha Diversity and operational taxonomic units (OTUs):.

Stage	Group Number	Group Name	OTUs	Chao1	Shannon	Gini–Simpson (%)	Good’s Coverage (%)
Sacrificed at week 5	1.	Male-C	238	250.05	5.23	93.42	99.89
	2.	Female-C	191	204.13	5.06	93.28	99.90
	3.	P-Male	157	172.33	4.34	90.32	99.84
	4.	P-Female	206	217.11	5.23	94.77	99.86
	5.	CPZ Male	192	234.27	5.01	92.70	99.82
	6.	CPZ Female	158	169.55	4.94	94.02	99.84
	7.	CPZ-Male-P	187	238.48	4.84	93.46	99.68
	8.	CPZ-Female-P	174	182.88	4.81	93.48	99.86
Sacrificed at week 7	9.	Male-C	187	200.8	3.15	69.29	99.91
	10.	Female-C	215	233.91	4.42	88.47	99.87
	11.	P-Male	238	281.04	4.93	93.20	99.79
	12.	P-Female	196	222.11	4.76	91.93	99.83
	13.	CPZ Male	161	190.18	4.27	90.65	99.83
	14.	CPZ Female	146	190	4.09	90.34	99.79
	15.	CPZ-Male-P	186	209.38	3.14	67.57	99.86
	16.	CPZ-Female-P	207	227.3	5.07	94.51	99.82

**Table 2:** Changes in the major microbial community at the *Phyla* during the demyelination and remyelination stages of MS in each group.

Group	Microbiome	Demyelination Stage	Remyelination Stage
Control	*Bacteroidetes*	28% in ♂—40.2% in ♀	Decreased in both sexes ^$^
	*Firmicutes*	38.6 in ♂—36.6% in ♀	Increased in both sexes ^$^
	*Proteobacteria*	19.2% in ♂—22.1% in ♀	A marked decrease in ♀ ^$^
*Palmaria p.*	*Bacteroidetes*	No marked change in both sexes *	Increased in both sexes *
	*Firmicutes*	Increased in both sexes *	Decreased in ♂ *
	*Proteobacteria*	Decreased in both sexes *	Decreased in ♀ > ♂ *
CPZ	*Bacteroidetes*	Increased in both sexes *	Increased in both sexes *
	*Firmicutes*	No marked change in both sexes *	Decreased in both sexes *
	*Proteobacteria*	++ decrease in both sexes *	Increased in both sexes *
CPZ + *Palmaria p.*	*Bacteroidetes*	Decreased in ♂ ** Increased in ♀ **	++ Increased in ♀ **
	*Firmicutes*	Increased in ♂ ** Decreased in ♀ **	++ Decreased in ♀ **
	*Proteobacteria*	Increased in ♀ > ♂ **	++ Increased in both sexes ^$^

* compared to control; ** compared to CPZ group; ^$^ compared to demyelination stage; ++ marked; ♀ female, ♂ male.

## Data Availability

All data are included in the manuscript, and no other data are available in other repositories.

## Supplementary Materials

The following supporting information can be downloaded at: https://www.mdpi.com/article/10.3390/ph16101355/s1. Figure S1: Open field test analysis of the total distance traveled (A) and velocity (B) of animals in both the demyelination and remyelination stages, Figure S2: Graph depicting Principal Coordinate analysis (Unweighted Unifrac). Samples are indicated with group number mentioned in Table 1, Figure S3: (A) A photomicrograph of a section in the brain of the CPZ group of adult males at the end of the 5th week showing a marked increase in the thinking of the corpus callosum, as well as an increase in the dense connective fibers. Notice marked vacuolated cytoplasm with cellular infiltration in the nerve cell process. (B) Notice marked vacuolated cytoplasm (black arrow) with cellular infiltration in the nerve cell process. Evidence of pyknotic nuclei (blue head arrow), also marked destructed oligodendrocytes (red arrow), and final appearance of demyelinated nerves (red arrow). (C) A photomicrograph of Section of the CPZ + *Palmaria P*. group at the end of the 5th of adult male showing corpus callosum with normally apparent Schwann cells (red arrow) with evidence of shown myelinated nerves (black arrow). H&E, × 400. Figure S4: (A) A photomicrograph of a section in the brain at the end of the 7th week of adult male mice showing apparent mild thinking of the CC (black arrow), as well as increasing the dense connective fibers. There is less vacuolated cytoplasm as well as a decrease in cellular infiltration (blue head arrow). (B) A photomicrograph of a section in the brain of adult Female mice at the end of the 7th week, showing mild thinking of the CC (black arrow), as well as an increase in the dense connective fibers. There is vacuolated cytoplasm. (blue head arrow), Notice there is a marked increase in cellular infiltration (redhead arrow). H&E, × 400.
