# Supplementary material for: Modulation of Gut Microbiome Community Mitigates Multiple Sclerosis in a Mouse Model: The Promising Role of Palmaria palmata Alga as a Prebiotic
Source: Pharmaceuticals (Basel). 2023 Sep 25;16(10):1355. doi: 10.3390/ph16101355 (PMC10610500; doi:10.3390/ph16101355)
Supplement: Supplementary file 1 [file pharmaceuticals-16-01355-s001.zip › pharmaceuticals-2595811-supplementary.pdf]

Supplementary Materials: Modulation of Gut Microbiome Community Mitigates Multiple Sclerosis in a Mouse Model: The Promising Role of *Palmaria palmata* Alga as a Prebiotic

Shimaa Mohammad Yousof, Badrah S. Alghamdi, Thamer Alqurashi, Mohammad Zubair Alam, Reham Tash, Imrana Tanvir and Lamis AbdelGadir Kaddam

A

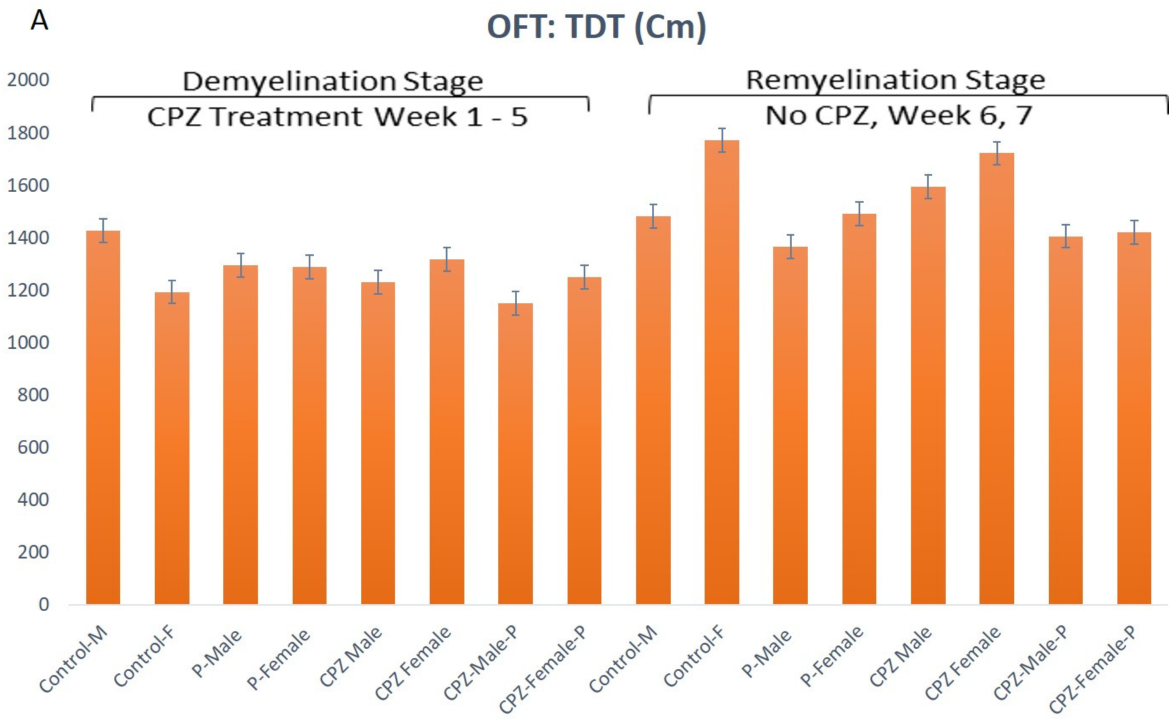

B

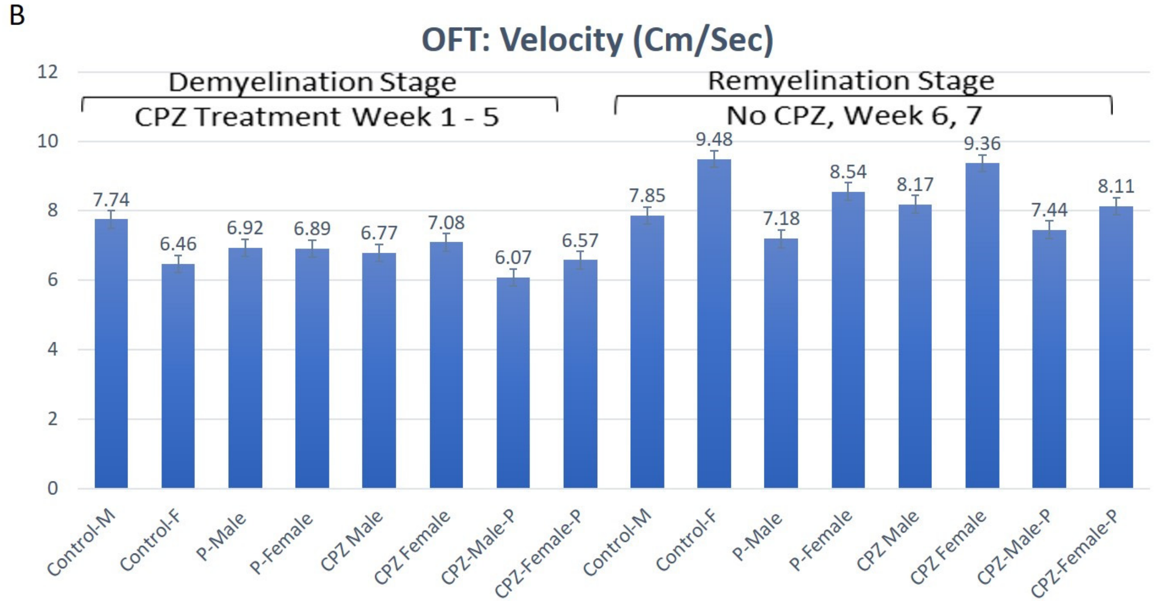

Figure S1. Open field test analysis of the total distance traveled (**A**) and velocity (**B**) of animals in both the demyelination and remyelination stages.

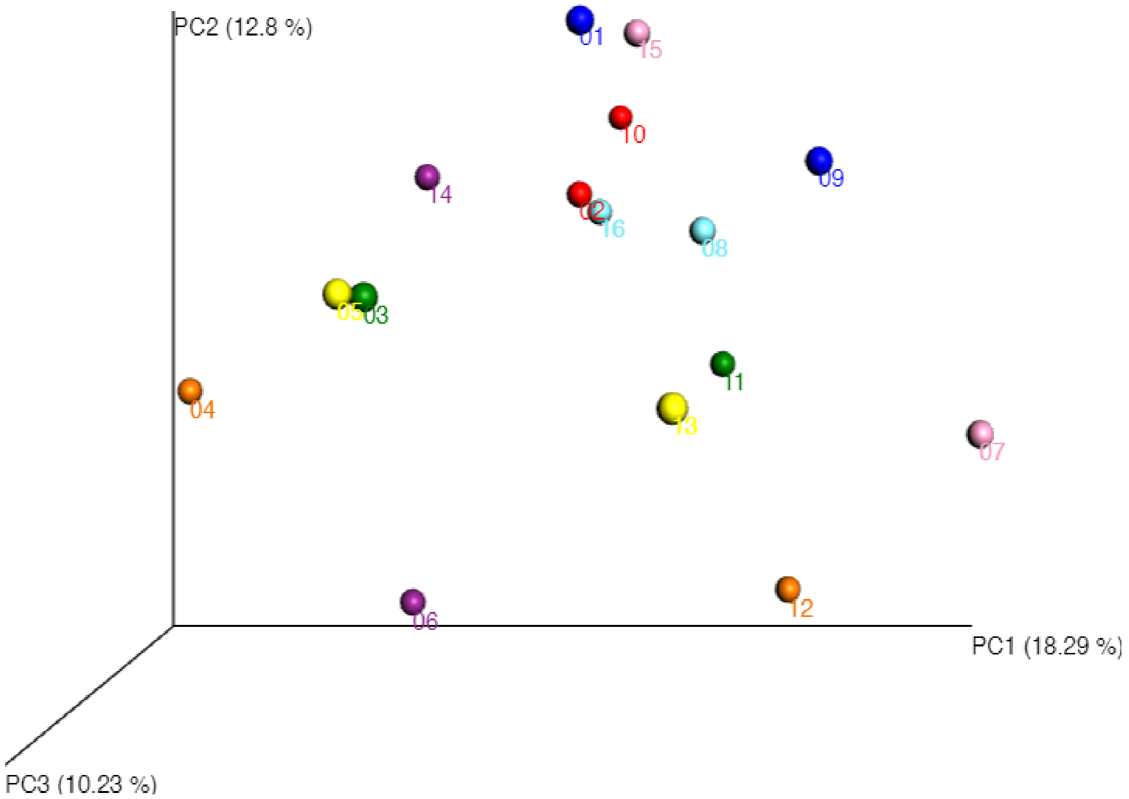

Figure S2. Graph depicting Principal Coordinate analysis (Unweighted Unifrac). Samples are indicated with group number mentioned in Table 1.

Male at 5th week (Demyelination stage) × 400

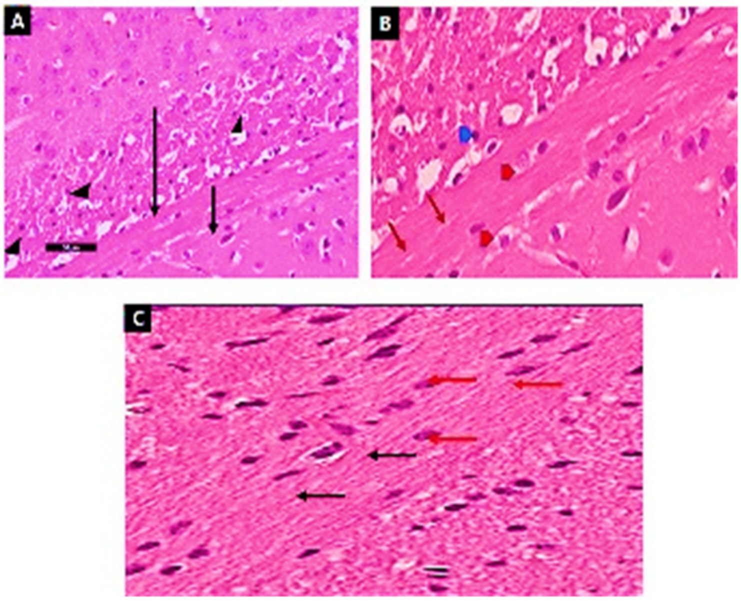

Figure S3. (**A**) A photomicrograph of a section in the brain of the CPZ group of adult males at the end of the 5th week showing a marked increase in the thinking of the corpus callosum, as well as an increase in the dense connective fibers. Notice marked vacuolated cytoplasm with cellular infiltration in the nerve cell process. (**B**) Notice marked vacuolated cytoplasm (black arrow) with cellular infiltration in the nerve cell process. Evidence of pyknotic nuclei (blue head arrow), also marked destructed oligodendrocytes (red arrow), and final appearance of demyelinated nerves (red arrow). (**C**) A photomicrograph of

Section of the CPZ + *Palmaria P*. group at the end of the 5th of adult male showing corpus callosum with normally apparent Schwann cells (red arrow) with evidence of shown myelinated nerves (black arrow). *H&E, × 400***.**

Male at 7th week (Remyelination stage) × 400 Female at 7th week (Remyelination stage) × 400

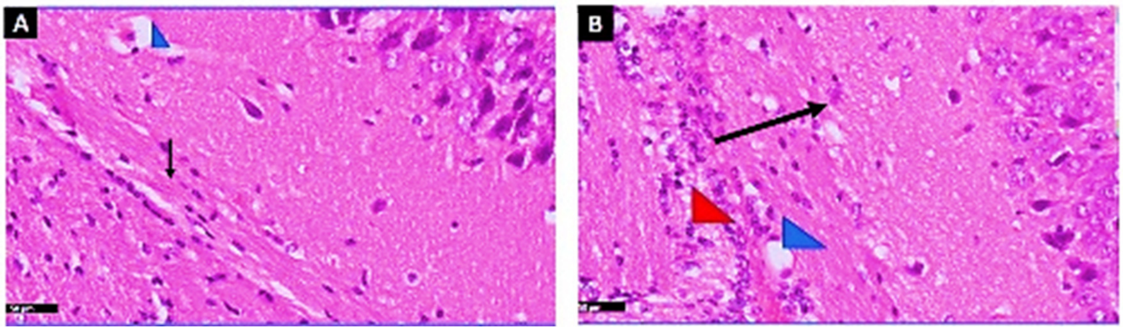

Figure S4. (**A**) A photomicrograph of a section in the brain at the end of the 7th week of adult male mice showing apparent mild thinking of the CC (black arrow), as well as increasing the dense connective fibers. There is less vacuolated cytoplasm as well as a decrease in cellular infiltration (blue head arrow). (**B**) A photomicrograph of a section in the brain of adult Female mice at the end of the 7th week, showing mild thinking of the CC (black arrow), as well as an increase in the dense connective fibers. There is vacuolated cytoplasm. (blue head arrow), Notice there is a marked increase in cellular infiltration (redhead arrow). *H&E, × 400.*
